# Efficiency and Security Evaluation of Lightweight Cryptographic Algorithms for Resource-Constrained IoT Devices

**DOI:** 10.3390/s24124008

**Published:** 2024-06-20

**Authors:** Indu Radhakrishnan, Shruti Jadon, Prasad B. Honnavalli

**Affiliations:** Department of Computer Science and Engineering, PES University, Bengaluru 560085, India; shrutijadon@pes.edu (S.J.); prasadhb@pes.edu (P.B.H.)

**Keywords:** LWC, IoT, benchmarking, resource-constrained devices, Arduino Nano, Arduino Micro

## Abstract

The IoT has become an integral part of the technological ecosystem that we all depend on. The increase in the number of IoT devices has also brought with it security concerns. Lightweight cryptography (LWC) has evolved to be a promising solution to improve the privacy and confidentiality aspect of IoT devices. The challenge is to choose the right algorithm from a plethora of choices. This work aims to compare three different LWC algorithms: AES-128, SPECK, and ASCON. The comparison is made by measuring various criteria such as execution time, memory utilization, latency, throughput, and security robustness of the algorithms in IoT boards with constrained computational capabilities and power. These metrics are crucial to determine the suitability and help in making informed decisions on choosing the right cryptographic algorithms to strike a balance between security and performance. Through the evaluation it is observed that SPECK exhibits better performance in resource-constrained IoT devices.

## 1. Introduction

In recent years, the adoption of the Internet of Things (IoT) has seen exponential growth in various domains including smart homes, healthcare, industrial systems, etc. This growth is fueled by the pervasive and ubiquitous nature of IoT sensors and devices, which serve as gateways between the digital world and the physical world. Additionally, the IoT has gained attention from both industry and academia due to its potential to revolutionize various sectors such as transportation, logistics, utilities, and mobile services [1]. The vast use cases of the IoT also come with a few innate challenges, such as low memory, low processing power, battery constraints, etc. One of the most critical concerns that many scientists are addressing is the security of IoT devices and their ecosystem. Ensuring the security and integrity of data transmitted between these devices has become a critical concern [2].

With the widespread use of IoT devices comes the need for robust security measures to protect the sensitive data transmitted and stored by these devices [3]. One of the main challenges in securing IoT devices is their resource-constrained nature, with limited memory and processing capabilities. Of all the security measures that have to be taken, using the right cryptographic algorithms is crucial in establishing the confidentiality, integrity, and authenticity of IoT data. It is also important to note that there is a need for efficient and robust cryptographic techniques to handle the security requirements.

The normal cryptographic algorithms tend to use a lot of resources, which could turn out be expensive in terms of resources, during their implementation in resource-constrained IoT devices. According to Nayak, Swapna [4], attention must be paid to the overhead complexity of network resources due to large-scale connectivity and group key management policies when considering using cryptographic algorithms for IoT applications. It is therefore suggested to use a group of cryptography algorithms called lightweight cryptographic algorithms (LWCs) that utilize low power and resources, thereby reducing overhead resource requirements for securing these devices efficiently and effectively [5].

In recent years, a lot of research has been conducted to optimize the performance of LWC algorithms for resource-constrained devices. Optimizing software and hardware for devices with limited resources involves tweaking both software and hardware features to optimize their efficiency, performance, and functionality. Stefano Di Matteo, Matteo Lo Gerfo et al., in their study [6], have shown how optimizing can significantly improve the performance, efficiency, and security of cryptographic algorithms. The goal of these initiatives is to guarantee strong security while addressing the resource limitations of IoT devices. To make these algorithms work well on them, researchers have tried to reduce their computational complexity and memory requirements. For instance, Gross et al., in their study [7], worked on optimizing ASCON for CAESAR, the Competition for Authenticated Encryption.

Furthermore, a lot of advancements in hardware acceleration techniques have been made to improve the overall performance of LWC algorithms on these resource-constrained devices.

The proposed paper evaluates the performance and efficiency of different LWC algorithms for IoT devices by benchmarking their performance in resource-constrained IoT boards. Benchmarking is a common practice in evaluating the performance and efficiency of algorithms. This practice involves measuring various metrics such as the execution time, memory usage, and power consumption of different algorithms to determine their suitability for resource-constrained IoT devices. Sufficient analysis is required to decide that a given LWC algorithm is suitable to be implemented on resource-constrained IoT devices [8].

The main objectives of this work are to:

- Set up a test-bed, implement the chosen algorithms, and record the values of the chosen performance metrics.
- Inspect and evaluate the results of the performance metrics.
- Help researchers to identify the right LWC algorithms for these resource-constrained IoT boards.

The rest of the paper is divided as follows: Section 2 gives a brief insight into the challenges faced while using traditional cryptographic algorithms in the IoT ecosystem and the advantages of using light weight cryptography for the same. Section 3 highlights some findings from the previous work of other researchers in the area. The section also draws attention to the motivation of this work, evaluating the efficiency and security of AES-128 [9], SPECK, and ASCON on the Arduino Nano and Micro. IoT applications make extensive use of the Arduino Nano and Micro, which provide practical platforms for assessing the efficacy of LWC algorithms. Though this study is partially conducted by some researchers, it is still beneficial to conduct a thorough comparative analysis that focuses specifically on the Arduino Nano and Micro in order to make informed decisions about which LWC algorithm is best suited for specific IoT applications. Section 4 describes the methods used to perform the analysis, the criteria for choosing the boards, and the algorithms for evaluation. In Section 5, the readings, comparisons, and observations are discussed.

## 2. Lightweight Cryptography

**Figure 1:**
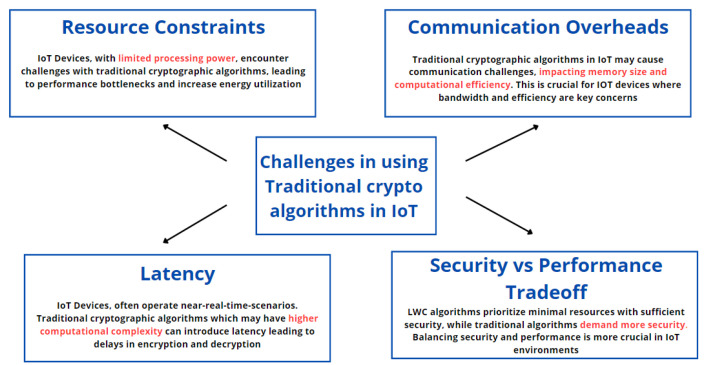
Challenges of using traditional cryptographic algorithms in IoT devices.

Most institutions and businesses are increasingly using the IoT to augment business process management and productivity. Seamless connectivity and interaction among IoT devices is achieved by peer-to-peer (P2P) communication. Also, IoT devices are often interconnected and capable of collecting sensitive data, resulting in them being a potential target for cyber attacks. Therefore, as the IoT ecosystem continues to grow and connect more and more devices, the need for robust security measures become increasingly important. The secure transmission of data between these devices is crucial to prevent unauthorized access and data breaches. Not taking necessary measures to protect the data at the endpoint devices could lead to loss of privacy and lead to unauthorized access to these sensitive data. This indicates that the encryption and decryption operations must be efficient, and utilize minimal computational power and resources. Among the many attack mitigation mechanisms, a significant technique is using the right cryptographic algorithms to secure the data transmitted from the endpoint devices to the cloud or the local aggregator. The key objective of cryptography is to convert the data into plaintext, to a scrambled form, using mathematical concepts such that any adversaries cannot interpret or manipulate the data without proper authorization and decryption keys. In the bargain of ensuring security, using a normal cryptographic algorithm in the IoT could prove to be resource consuming due to the intense computations required during the encryption and decryption process. Figure 1 encapsulates the challenges of using conventional cryptographic algorithms in the IoT.

Furthermore, one of the most critical issues identified in IoT systems is cryptographic failures which are often a result of using cryptographic algorithms that provide inadequate security for resource-constrained environments. This has been enumerated in the common weakness enumerations (CWEs) as CWE-327: broken or risky crypto algorithm [10]. Given the limited resources such as memory, processor capability, and battery constraints in IoT devices, it is crucial to use LWC algorithms that can provide adequate security without putting too much computational and resource overhead on the IoT devices [11]. LWC algorithms have a smaller memory footprint, lower computational requirements, and reduced power consumption, making them suitable for use in resource-constrained IoT applications.

The National Institute of Standards and Technology (NIST) hosted a lightweight cryptography workshop in 2015 to address the security and resource requirements that should be included in a standard to secure an IoT ecosystem. A total of 56 algorithm proposals, including more than 200 cipher implementation variants, were received and published by NIST [12]. SPN networks, hash functions, and authentication techniques customized for the target platforms are some frequently used structures by LWC algorithms.

A structural classification of LWC algorithms follows:

1. SPN networks: Substitution-permutation networks, known as SPN networks, are a highly optimized approach in LWC algorithms. This methodology involves dividing the input into blocks and performing sequential substitution and permutation operations. SPN networks are particularly effective for resource-constrained IoT devices due to their efficiency in terms of memory usage and computational resources.
2. Sponge-based: A sponge construction is like a random oracle, it takes a variable length input and gives an infinite length output. A high-level working of a sponge function is that it absorbs the input data, and then, squeezes out some data, just enough to form a hash [13]. Sponge construction was introduced as a component of the Keccak hash algorithm, which was the selected algorithm for in the next SHA-3 (Secure Hashing Algorithm-3) competition [14].
3. ARX ciphers: ARX ciphers are a family of symmetric-key cryptographic algorithms consisting of a combination of modular addition, bitwise rotation and XoR operations. ARX ciphers have gained a lot of traction to be used in IoT applications because of their simplicity, compactness, and ease of implementation [15].
4. AEAD ciphers: AEAD also known as authenticated encryption with associated data is a group of cryptographic algorithms that provides authentication and encryption. This is achieved by encrypting the plaintext, and then, authenticating internally using an authenticating tag. The advantage of this family of algorithms is that it ensures integrity of the communication while ensuring the size of the code is small and the implementations uses less computational time.

Table 1 summarizes the different algorithms that fall under each of the categories and their key features.

## 3. Literature Survey

In this section of the paper, we provide an overview of related work in the field of lightweight cryptography for IoT devices. Several studies have been conducted to evaluate and compare the performances of different LWC algorithms in terms of security, efficiency, and resource utilization. The challenge of selecting the right LWC algorithm arises due to the existence of diverse algorithms available for securing IoT devices. Some prominently used lightweight encryption techniques for IoT ecosystems are AES, SIMON, PRESENT, and ASCON, which have shown some promising outcomes with respect to efficiency, protection, and energy usage. While choosing a lightweight cryptographic algorithm for IoT applications, it is important to carefully assess various essential criteria to ensure that the appropriate algorithm aligns well with the specific needs and constraints of IoT devices. Some considerations to be made before choosing the right LWC algorithm for IoT applications include:

- The computational capabilities available for the IoT devices should be thoroughly evaluated before choosing an LWC algorithm.
- Security considerations and requirements required for the application.
- Performance evaluation, considering factors such as computational complexity, memory usage, energy consumption, security, and interoperability requirements.

By carefully evaluating these performance metrics, the most suitable algorithm can be selected to optimize the overall performance of the IoT system while satisfying the resource constraints. Therefore performance evaluation of LWC algorithms for IoT devices is crucial to ensure that they can effectively meet the specific needs and constraints of the device [16]. Saffer et al. [17] provide an analytical comparison of the most recent lightweight stream ciphers based on linear-feedback shift register (LFSR) and nonlinear-feedback shift register (NLFSR).

Extensive work has been performed to enhance the efficiency and practicality of cryptography on resource-constrained devices through considerable hardware and software modifications. Liu, T. et al. [18] have performed a consolidated analysis of the recent advancements in performance evaluation, software/hardware optimization, and GPU acceleration for post-quantum cryptography (PQC) in IoT applications. The research of Marin L. et al. [19] is focused on optimizing ECC algorithms for IoT devices based on NXP/Jennic 5148, a wireless microcontroller designed for use in low-power wireless applications. These algorithms might be used in conjunction with MSP430-optimized equivalents to provide secure communication in IoT networks. It is worthy to mention that while the implementation of both hardware and software optimizations in resource-constrained IoT devices is crucial for enhancing performance, energy efficiency, and reliability, these optimizations can sometimes lead to increased complexity and development costs, posing a challenge for widespread adoption and rapid deployment.

One important study in assessing the need of optimization is [20], in their work the authors evaluated hardware implementations of six selected candidates (SpoC, GIFT-COFB, COMET-AES, COMET-CHAM, ASCON, and Schwaemm and Esch) from round 2 of the NIST LWC algorithms. They emphasized the significance of early hardware implementation information in cryptographic challenges since certain ciphers showed better throughput and TPA ratios than others.

El-Hajj et al. [21] have performed a comprehensive analysis of the second-round NIST candidates (80 stream and block cipher algorithms) in terms of latency and energy efficiency, adding them to the initial set of ciphers. The analysis concentrates on assessing and comparing lightweight symmetric ciphers on particular non-resource-constrained (with respect to memory and CPU) reference platforms like Arduino and Raspberry, potentially restricting the broad applicability of the results to other resource-constrained IoT hardware platforms.

Abdel-Halim et al. [22] have evaluated the performance of lightweight block ciphers (GIFT-COFB, Romulus, and TinyJAMBU) on the Arduino Duo, which is a resource-constrained IoT device. However, the authors do not provide a comparison with other existing lightweight block ciphers or a benchmark against industry standards for performance evaluation. Adding to this, the authors do not consider the security aspects of the evaluated ciphers in detail, as the work mainly focuses only on the performance metrics.

Hasan et al. [23] have reviewed four lightweight cryptography (LWC) algorithms proposed in the ISO/IEC 29192 standard [24]: SIMON, SPECK, PRESENT, and CLEFIA, focusing on their security and performance aspects for IoT-enabled devices. The aim of this study was to identify LWC algorithms that are optimized for IoT applications while still providing adequate security levels. The authors do not provide a comparative analysis of the reviewed algorithms, making it difficult to determine which algorithm may be more suitable for specific IoT applications.

Regla et al. [25] performed a systematic review of the literature to identify lightweight cryptography algorithms and extract relevant data such as level of security, encryption and decryption performance, execution time, memory usage, clock speed, latency, and frequency. The study provides insights into the performance of lightweight cryptography algorithms in IoT devices, helping researchers understand their strengths and weaknesses. Nevertheless, the paper does not discuss the potential trade-offs between security and performance in the context of lightweight cryptographic algorithms for IoT applications.

Many researchers have conducted a variety of studies to evaluate the performances of various LWC algorithms for IoT devices. These studies have focused on factors such as code size, RAM consumption, and execution time of encryption, decryption, and key scheduling. The studies have been conducted on hardware devices, some with good computational resources while others have limited resources.

However, in [26], the authors Damaj et al. proposed a unified analytical framework for evaluating lightweight cryptographic algorithms in heterogeneous computing environments, considering both hardware and software metrics. The framework suggested in the study utilizes three decision-making approaches TOPSIS, PROMETHEE II, and Fuzzy TOPSIS, to enable effective evaluations and adjustments to cryptographic algorithm implementations. However, the authors do not discuss the specific LWC algorithms that were evaluated using the proposed framework.

Another study, by Sevin et al. [27], compares more than 50 LWC algorithms and their performances. However, the authors do not explicitly mention the specific IoT platform used for the implementation of lightweight block ciphers. This work focuses its analysis on a software platform with an 8-bit architecture, which may not encompass all potential IoT platforms.

LWC algorithms’ simplicity and low multiplicative depth contribute to their efficiency and suitability for constrained platforms, including ASICs, FPGAs, and microcontrollers [28].

Jangra et al. [29] have performed an evaluation of SPECK in comparison with another LWC called SIMON. The evaluation highlights the suitability of SPECK as a better option for securing smart city applications on a Raspberry Pi.

Another crucial factor is to consider the cryptographic properties and features of the algorithms such as security strength, key sizes, resistance to potential attacks, and the trade-offs between security and performance. The evaluation should also assess their suitability for resource-constrained IoT devices [30].

An important observation from the literature survey is that the evaluations have either been performed on simulators or on IoT devices with good computational and memory resources. The evaluations have not been performed on resource-constrained devices which are commonly used; thus providing a motivation for our research.

## 4. Methods

In this section, we describe the selection criteria for the boards and algorithms used, the experimental setup, and the evaluation mechanism used.

### 4.1. Overview

The goal of this study is to evaluate the performance of existing LWC algorithms on resource-constrained boards for IoT devices. The evaluation will focus on several key factors, including memory consumption, speed, throughput, energy efficiency, and scalability. The evaluation is performed by setting up a test-bed that includes representative IoT devices and running a series of tests by implementing the selected LWC algorithms on these devices. To perform this evaluation, we have shortlisted three LWC algorithms that are NIST-approved and commonly used in IoT applications: AES-128, SPECK, and ASCON. Based on the evaluation, benchmarks are conducted to analyze the performance of these algorithms in terms of memory consumption, speed, throughput, and energy efficiency. This evaluation is crucial in order to determine their suitability and effectiveness in meeting the specific needs and constraints of resource-constrained devices. The results of the evaluation will provide valuable insights into the strengths and weaknesses of each algorithm, allowing IoT developers to make informed decisions regarding algorithm selection for their specific application needs in resource-constrained environments.

### 4.2. Selection of Boards

The first step in this study is to identify resource-constrained boards that are commonly used for IoT devices. Resource-constrained boards are important for IoT applications as they enable low power consumption, low maintenance costs, and longer battery life [31]. These boards are used in various domains and require seamless integration among heterogeneous objects. However, monitoring and tracking the energy consumed during the software implementation and ensuring secure and efficient implementation of these cryptographic algorithms is challenging due to the limited resources of these boards [32].

Table 2 is a list of boards that are resource-constrained and commonly used to develop IoT applications.

**Figure 2:**
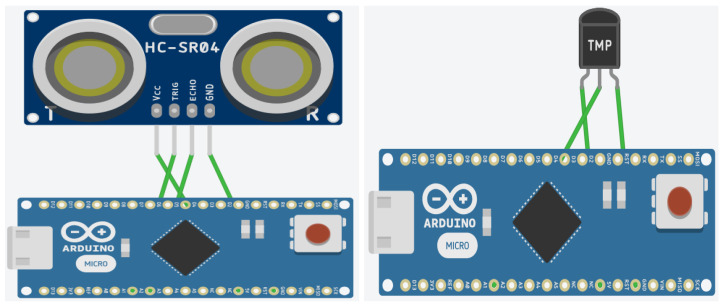
Test-bed setup.

Of all the available IoT boards listed in Table 2, we have shortlisted the Arduino Nano running on an ATmega328P microcontroller, with 32 KB flash and 2 KB RAM, and the Arduino Micro running on an ATmega32U4 microcontroller, with 32 KB flash and 2.55 KB RAM. The boards mentioned above were selected due to their widespread acceptance in the IoT field and the fact that they are common to a lot of IoT devices with limited resources, therefore they offer a practical foundation for evaluating the effectiveness and security of lightweight cryptographic techniques. Additionally, they are capable of supporting lightweight cryptography algorithms. The test-bed setup is as depicted in Figure 2.

### 4.3. Selection of Algorithms

A lot of LWCs have been proposed for resource-constrained IoT devices, each with its own unique design and cryptographic properties. It is imperative to select algorithms that strike a balance between security, efficiency, and compatibility with IoT hardware platforms. Moreover, it is crucial to consider the cryptographic properties and features of the algorithms such as security strength, key sizes, resistance to potential attacks, and the trade-offs between security and performance. The evaluation should also assess the algorithms’ resistance to side-channel attacks and their suitability for constrained IoT devices [33]. Choosing three algorithms suitable for resource-constrained boards from a plethora of available algorithms is a challenging task given the heterogeneous nature of IoT devices and the diverse requirements of IoT applications. Below are some tables consolidating the different LWC algorithms classifying them according to the structure they belong to and providing some description. There are many such algorithms that fall under each of the categories, of which the listed ones are popularly used.

Table 3 gives an insight into some LWC algorithms based on SPN structures with their key size and number of rounds in the algorithm, with AES-128 and PRESENT being some commonly used algorithms from this category.

Table 4 provides an overview of some LWC algorithms based on a Feistel network, generally used to optimize the security and efficiency of IoT devices.

Table 5 gives a comprehensive list of some popularly used LWC algorithms based on authenticated encryption to ensure data integrity and confidentiality in IoT devices.

Table 6 gives an overview of some popularly used LWC algorithms based on hashing that have minimal computational overhead.

Table 7 outlines some LWC algorithms based on add–rotate–XOR networks.

In this study, we have shortlisted three LWC algorithms for evaluation based on their performance, security, and compatibility with resource-constrained IoT devices. Table 8 summarizes the algorithms selected and their key features. The first algorithm selected for evaluation is ASCON, which has already been briefly introduced earlier in this paper. Its focus on lightweight implementation, robust data integrity, and NIST approval makes it a strong contender for IoT applications. The second algorithm chosen for evaluation is SPECK, known for its simplicity and efficiency in hardware implementations, making it a popular choice for lightweight cryptography in constrained environments. Finally, the third algorithm to be evaluated is AES-128, which, although not specifically designed for lightweight cryptography, has been adapted and optimized for resource-constrained IoT devices and has a proven track record of security.

AES-128: AES-128, also known as Advanced Encryption Standard with a 128-bit key length, is a lightweight version of the AES encryption algorithm. It is widely recognized for its robust security and efficient handling of memory consumption, speed, and throughput. AES is a substitution-permutation network algorithm that operates on blocks of data to provide secure encryption [34]. AES-128 operates on a 128-bit block of data and uses a 128-bit key for encryption. During the encryption process, AES-128 applies a series of substitution and permutation operations to the input data, followed by multiple rounds of mixing the data through linear and nonlinear transformations. This process results in a cipher text that is resistant to various types of cryptographic attacks.

SPECK: SPECK is another lightweight cryptographic algorithm commonly used in IoT applications. It is known for its simplicity and compactness, making it suitable for resource-constrained IoT devices. SPECK is based on ARX operations, which makes it efficient in terms of memory consumption and execution time [35]. SPECK comes in different configurations with varying block sizes and key sizes. The most popular versions are SPECK32/64, SPECK48/72, and SPECK64/128, where the numbers represent the block size and key size in bits [36]. It is based on a Feistel network structure in which each block is divided into two halves, each of the blocks then undergoes a sequence of operations using sub-keys derived from the original key. These operations include bitwise XOR, rotation, and modular addition within each round to enhance the confusion and diffusion properties of the cipher.

ASCON: ASCON is a NIST-approved algorithm designed specifically for providing secure and efficient encryption on constrained devices in IoT applications. It offers lightweight implementation while ensuring data integrity and confidentiality. ASCON is a sponge-based AEAD algorithm, meaning it provides both encryption and authentication of the data [37]. Because of its lightweight and effective security features, it can be used in a wide range of applications as a part of the Internet of Things (IoT). ASCON uses a sponge construction based on permutations. This method processes input data in blocks using a specific permutation, and operates on fixed-size blocks with a set output length. The sponge construction is a versatile framework that can be used for various cryptographic purposes. It involves absorbing input data into the internal state, followed by squeezing the output from the state.

These three algorithms represent a diverse range of cryptographic designs and properties, allowing for a comprehensive evaluation of their performance on resource-constrained IoT devices. Also, analyzing AES-128, SPECK, and ASCON provides a comprehensive view of the trade-offs between security and performance, helping to determine the most suitable cryptographic solution for resource-constrained IoT devices.

AES-128 is a widely adopted encryption standard, offering robust security and resistance to cryptographic attacks and also serving as a benchmark to compare against newer, lightweight algorithms and provides a baseline for security and performance. On the other hand, designed specifically for lightweight applications, SPECK aims to balance security and efficiency. Comparing SPECK’s performance against AES-128 demonstrates how a lightweight approach can mitigate the challenges posed by resource-constrained devices. Including ASCON in the analysis demonstrates how modern cryptographic solutions can achieve a high level of security with optimized performance, providing a comprehensive understanding of the trade-offs between security and efficiency and identifying the most suitable cryptographic solution for resource-constrained IoT devices.

Table 8 gives a comprehensive outline of the characteristics of the chosen algorithms for the study.

In the upcoming sections, the evaluation results and analysis of these algorithms will provide valuable insights to aid IoT developers in selecting the most suitable algorithm for their specific application needs.

### 4.4. Evaluation Methods

In order to evaluate the performance and benchmark the selected lightweight cryptography algorithms, a comprehensive test-bed was set up. The chosen LWC algorithms were meticulously implemented on specific boards, and an array of parameters, as mentioned earlier, were systematically measured and critically compared. Different sensors were used in the test-bed to measure the parameters so that the readings are not biased towards a certain type of sensor or a certain type of IoT use case.

The chosen algorithms were implemented in the C language and optimized for resource-constrained IoT devices using efficient coding techniques such as minimizing memory usage, reducing computational complexity, and optimizing operations for low-power consumption. These implementations were optimized to be used with the Arduino Nano and Arduino Micro boards using the Arduino IDE, Version: 2.1.1.

## 5. Results and Discussions

In this section of the paper, the evaluation criteria, readings, and inference from the experimental setups are discussed.

### 5.1. Evaluation Criteria: Metrics for Performance Evaluation

To evaluate the performance of the selected LWC algorithms, we will consider the following metrics:

1. Memory utilization: Since the algorithms are implemented in a resource-constrained environment, memory utilization plays a significant role in their feasibility and practicality. This metric measures the size of the algorithm’s code in rest (ROM utilization), which includes all the necessary operations for encryption and decryption and RAM utilization. In addition to this, it also accounts for any auxiliary functions or subroutines utilized within the algorithmic process [38]. Memory utilization is measured in two parts:
  (a) Code size/ROM utilization: Resource-constrained IoT boards have small amounts of flash memory, which serves as both program storage (including code and read-only data) and non-volatile storage for configuration data or persistent state. Code size or ROM utilization is the size of the executable that is programmed into the ROM/flash memory of the boards.
  (b) RAM utilization: This is a measure of the amount of random access memory (RAM) utilized by the algorithm during its execution, allowing for a measurement of resource efficiency and potential performance limitations [39]. Additionally, power consumption is another metric that is considered in the evaluation process [40].

**Figure 3:**
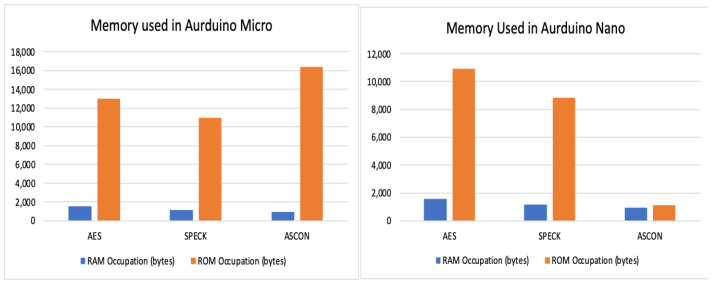
Memory used in Arduino Nano and Micro.

Observations and evaluation of RAM and ROM occupation:

Table 9 and Table 10 illustrate the RAM and ROM occupation of the algorithms during execution. Figure 3 shows a graphical comparison of the same.

ASCON has the smallest RAM utilization, making its implementation lightweight in terms of memory used at rest and the SPECK implementation has the smallest ROM utilization size of all the three algorithms, making its implementation lightweight in terms of memory used at run time.

2. Execution time (encryption and decryption speed): This metric measures the time taken by the algorithm to perform encryption and decryption operations [41]. The measurements do not consider the main function’s overhead and focus solely on the cryptographic operations themselves. The encryption and decryption speeds are measured by executing the algorithms with different input sizes and recording the time taken for each operation [42].
  (1) Executiontime=t1+t2+t3:
  where *t*1 = encryption time; *t*2 = decryption time; and *t*3 = time taken for key generation.

**Figure 4:**
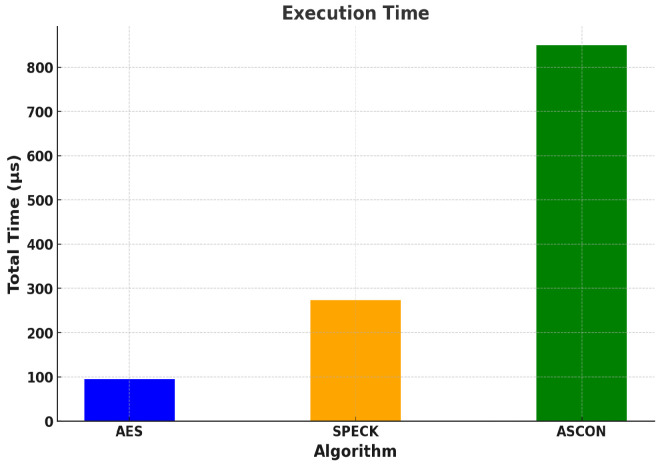
Execution times of AES-128, SPECK, and ASCON.

Observations and evaluation of execution time:

Table 11 shows the readings of the observation of execution times of the different algorithms. These reading are the same for both the boards. AES-128, of all the three algorithms, is observed to be the fastest. The simple steps during its encryption and decryption contribute to the speed of execution. Figure 4 shows a graphical representation of the same.

3. Throughput: Throughput is a measure of the efficiency and speed of cryptographic algorithms, indicating the number of encryption or decryption operations that can be executed within a given time. The throughput of an encryption scheme depends on factors like complexity of the algorithm, the processing power of the hardware used, and if any optimization mechanisms are used in the software or the hardware. In the case of cryptographic algorithms, the key scheduling process can also have an impact on the throughput of the algorithm.
  (2) Throughput=α/t
  where α = number of bits; *t* = time taken for the operation.

**Figure 5:**
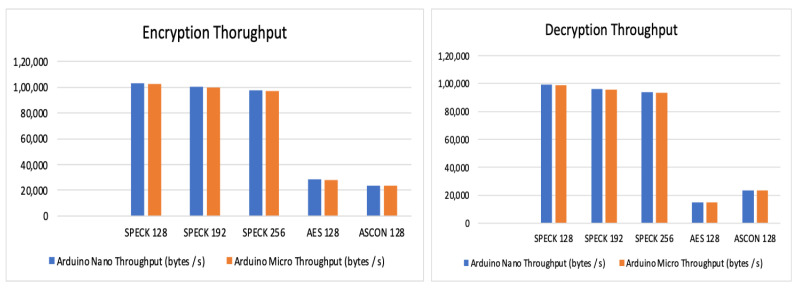
Encryption and decryption throughput.

Observations and evaluation of throughput:

The throughput is measured during encryption and decryption separately. Table 12 and Table 13 summarize the observation of the throughput measured. Figure 5 shows a graphical comparison of the same. The observation is that SPECK exhibits the highest throughput of the three algorithms.

4. Speed latency: The speed latency metric measures the time delay between initiating a cryptographic operation and receiving the output result [43].
  Speed latency = time for one operation to complete/number of operations performed.
  (3) SpeedLatency=Throughput/β
  where β = number of operations performed.

**Figure 6:**
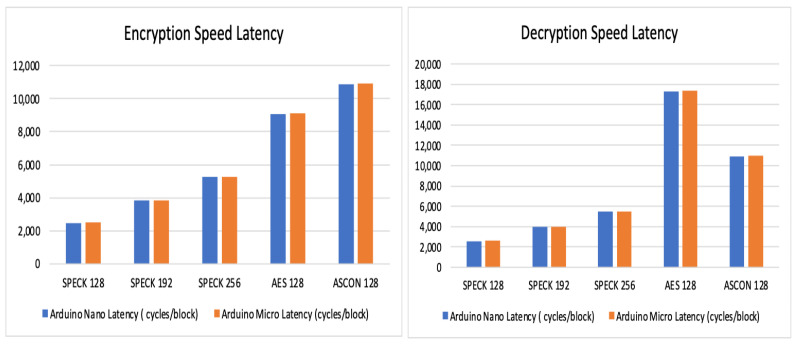
Encryption and decryption latency.

Observations and readings of speed latency:

The readings in Table 14 and Table 15 provide an insight into the speed latency of the three algorithms, supporting these tables is the graphical representation in Figure 6. SPECK exhibits the least latency among the three algorithms, making it the most efficient one in terms of latency.

5. Key schedule speed: The key schedule speed metric measures the efficiency of the algorithm’s key generation process, which is essential for encryption and decryption operations. Key schedule speed = time taken for key generation/number of encryption or decryption operations performed [44].
  (4) Speed=γ/λ
  where γ = time taken for key generation; λ = number of encryption or decryption operations performed.

**Figure 7:**
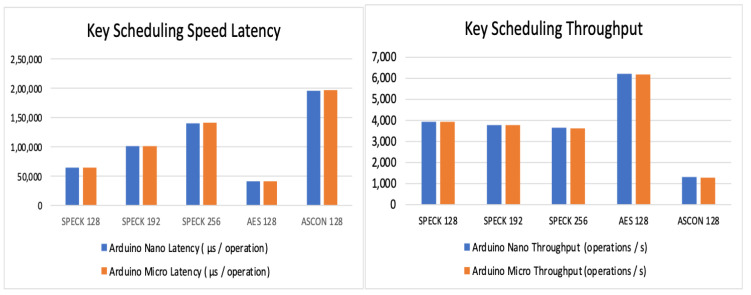
Key scheduling throughput and latency.

Observations of key schedule speed:

Table 16 and Table 17 show the values of the key scheduling speed throughput and latency of the three chosen algorithms and Figure 7 substantiates the understanding of the observations.

Having discussed the various metrics used to evaluate cryptographic algorithms, it is important to note that different algorithms may excel in different aspects.

### 5.2. Security Analysis

The security analysis of AES-128, SPECK, and ASCON for resource-constrained devices highlights the different trade-offs in terms of security and efficiency. Many researchers have conducted and provided a detailed security analysis of the chosen algorithms. The following is a comparative analysis of studies conducted.

- AES-128 (Advanced Encryption Standard): The NIST developed the symmetric encryption algorithm AES-128 (Advanced Encryption Standard). It has a strong defense against recognized cryptographic attacks, such as differential and linear cryptanalysis, making it a well-trusted security solution. On the other hand, the emergence of quantum computing presents symmetric-key algorithms with a theoretical threat. The studies suggest that employing Grover’s algorithm to speed up the brute force queries would result in a reduction in the effective key size for AES-128 to 2^64^.
- SPECK is a family of lightweight block ciphers, known for its simplicity and flexibility. Many studies have been performed on SPECK’s defenses against common cryptographic threats. While it shows some resistance, it is usually thought to be less safe than more complicated algorithms like AES. However, the algorithms should be implemented carefully to protect the applications against side-channel attacks [45].
- An exhaustive analysis of the security of ASCON in resource-constrained environments has been conducted by various researchers. It is observed that ASCON provides robust security guarantees against both confidentiality and integrity attacks [46]. It ensures encryption and authentication with low overhead, maintaining device performance and using minimum power consumption while maintaining high security standards.

Table 18 outlines the key comparisons of the three algorithms for resource-constrained devices, along with their security and efficiency metrics.

Based on the evaluation and benchmarking of the lightweight cryptography algorithms, it can be concluded that AES-128, SPECK, and ASCON have shown promising performance while meeting the evolving resource constraints of IoT devices. Analyzing the readings of throughput and speed latency during encryption and decryption reveals that SPECK demonstrates superior performance because of its higher throughput and lower latency. However, the key scheduling throughput and latency of AES-128 seem to be better compared to the rest. On the other hand, ASCON demonstrates high energy efficiency and minimal memory usage combined with strong resistance to cryptographic attacks, making it an ideal option to be used with IoT devices with limited resources with a trade-off between encryption and decryption speed.

## 6. Conclusions

The evaluation and benchmarking process herein has provided valuable insights into the suitability of lightweight cryptography algorithms for resource-constrained IoT devices. It can be suggested that while SPECK is a good choice for scenarios where high throughput and low latency are crucial and energy availability is not a challenge for IoT devices, AES-128 and ASCON can be considered for their energy efficiency and minimal memory usage. While AES is a well-tested algorithm which has undergone analysis by the cryptographic community, it involves complex mathematical operations, including multiple rounds of substitution, permutation, and mixing. These operations require significant processing power, which could turn out to be expensive due to the limited CPU capabilities of the chosen resource-constrained IoT devices. The additional latency introduced during the encryption and decryption is also due to its complexity. For IoT applications that require real-time or near-real-time responses, this added delay can be detrimental. However, SPECK is relatively new and its security, like other algorithms, is dependent on its implementation. ASCON, on the other hand, offers compelling competition to AES in terms of secure implementation and trust from the cryptographic community. It is recommended to consider the evaluation and determine its suitability within the specific context of an IoT deployment, taking into account factors like threat models, resource availability, and performance requirements.

## Figures and Tables

**Table 1:** Summary of the types of LWC algorithms and their key features.

Algorithm Type	Example Algorithms	Key Features	Characteristics
ARX	Salsa20, ChaCha, HIGHT, TEA	Simplicity: Basic operations like addition, rotation, and XOR.	Lightweight: Well suited for resource-constrained environments. Lack of permutation: May lack the confusion introduced by substitution-permutation networks.
AEAD	AES-GCM, ChaCha20-Poly1305, OCB	Authenticated encryption: Provides both confidentiality and integrity protection. Associated data: Allows for the authentication of additional associated data.	Online operation: Supports processing data in a streaming or online fashion. Parallelizable: Encryption and authentication operations can be parallelized.
SPN	AES (Rijndael), DES, PRESENT, SPECK	Confusion and diffusion: Achieves confusion through substitution and diffusion through permutation. S-box operations: Uses substitution boxes for nonlinear transformations.	Block size: Typically operates on fixed-size blocks of data, e.g., 128 or 64 bits. Feistel structure: Some SPN designs use a Feistel network structure.
Sponge-based	Keccak, SHA-3, Kangaroo, Twelve, NORX	Sponge construction: Absorbs input, squeezes output, and has a flexible capacity. Resistance to cryptanalytic attacks: Designed with a focus on resistance against various attack types.	Customizable parameters: Allows for tuning security levels and performance. Keccak permutation: Based on Keccak permutation, designed for cryptographic strength and flexibility.

**Table 2:** Table summarizing the commonly used resource-constrained boards for the IoT.

Board	Features and Notes	Specifications
Arduino Nano	Compact, cost-effective	ATmega328P microcontroller, 32 KB flash, 2 KB RAM
ESP8266	Low-cost Wi-Fi module	Tensilica L106 32-bit MCU, 4 MB flash, Wi-Fi
Arduino Micro	Small form factor, suitable for space-constrained projects	ATmega32U4 microcontroller, 32 KB flash, 2.5 KB RAM
Raspberry Pi Zero	Small form factor of Raspberry Pi	Broadcom BCM2835, 1 GHz ARM11, 512 MB RAM, HDMI, USB
ESP32	Wi-Fi and Bluetooth capabilities, more GPIO pins	Dual-core Tensilica LX6, 240 MHz, Wi-Fi, Bluetooth, 520 KB RAM
Particle Photon	Small Wi-Fi-enabled board with cloud connectivity	STM32F205, Wi-Fi, 1 MB flash, 128 KB RAM
MicroPython Boards	Runs MicroPython, efficient Python 3 for microcontrollers	Varies depending on the specific MicroPython board
NodeMCU	ESP8266-based, Lua scripting support	ESP8266, 4 MB flash, Wi-Fi

**Table 3:** LWCs based on substitution-permutation network (SPN).

Algorithm Name	Description	Key Size	Number of Rounds
Skinny	Block cipher designed for constrained environments	64/128	40
ACORN	Authenticated cipher for lightweight applications	80/128	160
LED	Lightweight block cipher	64/128	48
PRESENT	Lightweight block cipher	80/128	31
Hummingbird	Authenticated encryption algorithm	80/128	1–3
TWINE	Lightweight block cipher	80/128	36
AES-128	Advanced Encryption Standard	128	10

**Table 4:** LWCs based on Feistel network.

Algorithm Name	Description	Key Size	Number of Rounds
HIGHT	Block cipher optimized for hardware	64/128	32
SIMON	Lightweight block cipher	64/128/192/256	32/36/42/44

**Table 5:** LWC based on authenticated encryption.

Algorithm Name	Description	Key Size	Number of Rounds
MORUS	Authenticated encryption algorithm	128/256	12
LEDA	Authenticated encryption algorithm	64 to 128 bits	May vary
ASCON	Authenticated encryption algorithm and sponge based	128/256	12

**Table 6:** LWCs based on hashing.

Algorithm Name	Description	Key Size	Number of Rounds
PICNIC	Ultra-lightweight cryptographic hash function	128	Not applicable
BLAKE2	Lightweight cryptographic hash function and Merkle–Damgård construction	1–64 bytes	May vary

**Table 7:** LWCs based on add–rotate–XOR networks.

Algorithm Name	Description	Key Size	Number of Rounds
SPECK	Ultra-lightweight block cipher	64/128	22/26
PRINCE	Lightweight encryption algorithm	128, 192, or 256 bits	12

**Table 8:** Shortlisted algorithms for evaluation.

Algorithm Name	Description	Structure/Type	Block Size	Key Size	Number of Rounds
AES-128	Advanced Encryption Standard (AES)	Substitution-permutation network (SPN)	128 bits	128	10
SPECK	Ultra-lightweight block cipher	Add–rotate–XOR network	64 bits	64/128	22/26
ASCON	Authenticated encryption algorithm	Sponge construction	64 bits	128/256	12

**Table 9:** Memory occupation of LWC algorithm in Arduino Nano.

	RAM Occupation (Bytes)	ROM Occupation (Bytes)
AES	1570	10,928
SPECK	1164	8848
ASCON	940	1108

**Table 10:** Memory occupation of LWC algorithm in Arduino Micro.

	RAM Occupation (Bytes)	ROM Occupation (Bytes)
AES	1535	13,014
SPECK	1129	10,940
ASCON	905	16,354

**Table 11:** Execution times of LWC algorithms.

Algorithm	Encryption Time	Decryption Time	Key Generation Time	Total Time
AES	42.39 μs	42.56 μs	9.98 μs	94.93 μs
SPECK	9.74 μs	10.12 μs	253.87 μs	273.73 μs
ASCON	42.48 μs	42.67 μs	764.52 μs	849.67 μs

**Table 12:** Encryption throughput.

	Arduino Nano	Arduino Micro
**Encryption**	**Throughput (Bits/s)**	**Throughput (Bits/s)**
AES-128	226,041	224,911
SPECK 128	824,041	820,268
SPECK 192	800,700	797,027
SPECK 256	778,653	775,062
ASCON 128	188,349	187,381

**Table 13:** Decryption throughput.

	Arduino Nano	Arduino Micro
**Decryption**	**Throughput (Bits/s)**	**Throughput (Bits/s)**
AES-128	118,601	117,999
SPECK 128	793,214	789,573
SPECK 192	770,694	767,139
SPECK 256	749,418	745,955
ASCON 128	187,516	186,553

**Table 14:** Encryption latency.

	Arduino Nano	Arduino Micro
**Encryption**	**Latency (Cycles/Block)**	**Latency (Cycles/Block)**
AES-128	9060.301616	9105.806268
SPECK 128	2485.311904	2496.742531
SPECK 192	3836.639871	3854.32305
SPECK 256	5260.364408	5284.735784
ASCON 128	10,873.40175	10,929.57299

**Table 15:** Decryption latency.

	Arduino Nano	Arduino Micro
**Decryption**	**Latency (Cycles/Block)**	**Latency (Cycles/Block)**
AES-128	17,267.9418	17,355.97927
SPECK 128	2581.900715	2593.804799
SPECK 192	3986.015728	4004.486276
SPECK 256	5465.574619	5490.944018
ASCON 128	10,921.69274	10,978.1064

**Table 16:** Key scheduling throughput.

	Arduino Nano	Arduino Micro
**Key Scheduling**	**Throughput (Bits/s)**	**Throughput (Bits/s)**
AES-128	49,708	49,465
SPECK 128	31,575	31,417
SPECK 192	30,311	30,160
SPECK 256	29,151	29,006
ASCON 128	10,463	10,416

**Table 17:** Key scheduling latency.

	Arduino Nano	Arduino Micro
**Key Scheduling Latency**	**Latency (Cycles/Block)**	**Latency (Cycles/Block)**
AES-128	41,200.74419	41,402.91179
SPECK 128	64,861.03018	65,185.7284
SPECK 192	101,347.8739	101,855.9534
SPECK 256	140,506.482	141,210.9946
ASCON 128	195,720.1508	196,617.5635

**Table 18:** Security efficiency analysis.

Algorithm	Security Strength	Efficiency	Suitability for IoT Devices
AES-128	High	Moderate (computationally intensive)	Secure but resource-intensive
SPECK	Moderate (some scrutiny on security)	High (designed for lightweight use)	High (efficient for constrained devices)
ASCON	High (strong resistance to attacks)	High (optimized for performance)	High (low overhead, suitable for constrained devices)

## Data Availability

Data are contained within the article.

## Abbreviations

The following abbreviations are used in this manuscript:

LWC	Lightweight cryptography
P2P	Peer to peer
IoT	Internet of Things
NIST	National Institute Of Standards and Technology
AES	Advanced Encryption Standard
CWEs	Common weakness enumerations
SPN	Substitution-permutation network
PQC	Post-quantum cryptography
